# Long-Term Outcomes of Caustic Esophageal Stricture with Endoscopic Balloon Dilatation in Chinese Children

**DOI:** 10.1155/2018/8352756

**Published:** 2018-08-08

**Authors:** Lan-Lan Geng, Cui-Ping Liang, Pei-Yu Chen, Qiang Wu, Min Yang, Hui-Wen Li, Zhao-Hui Xu, Lu Ren, Hong-Li Wang, Shunxian Cheng, Wan-Fu Xu, Yang Chen, Chao Zhang, Li-Ying Liu, Ding-You Li, Si-Tang Gong

**Affiliations:** ^1^Department of Gastroenterology, Guangzhou Women and Children's Medical Center, 9 Jinsui Road, Guangzhou 510623, China; ^2^Division of Gastroenterology, Children's Mercy Hospital, 2401 Gillham Road, Kansas City, MO 64108, USA

## Abstract

Caustic esophageal stricture (CES) in children still occurs frequently in developing countries. We aimed to evaluate the long-term outcomes of endoscopic balloon dilatation (EBD) in treating CES in children and the influencing factors associated with outcome. We retrospectively reviewed the data of all patients who had a diagnosis of CES and underwent EBD from August 1, 2005, to December 31, 2014. The primary outcome was EBD success, which was defined as the maintenance of dysphagia-free status for at least 12 months after the last EBD. The secondary outcome was to analyze influencing factors associated with EBD success. Forty-three patients were included for analysis (29 males; mean age at first dilatation 44 months with range 121 months). 26 (60.5%) patients had long segment (>2 cm) stricture. A total of 168 EBD procedures were performed. Twenty-six (60.5%) patients were considered EBD success. Seventeen (39.5%) patients failed EBD and required stent placement and/or surgery. Patients in the EBD success group had significantly shorter stricture segments when compared to the EBD failure group (*t* = 2.398, *P* = 0.018, OR = 3.206, 95% OR: 1.228–8.371). Seven (4.4%) esophageal perforations occurred in 6 patients after EBD. Stents were placed in 5 patients, and gastric tube esophagoplasty was performed in 14 patients. In conclusion, 26 (60.5%) of 43 children with CES had EBD success. Length of stricture was the main influencing factor associated with EBD treatment outcome.

## 1. Introduction

Caustic esophageal stricture (CES) in children still occurs frequently in developing countries, due to accidental ingestion of caustic substances including strong alkalis and acids [1, 2]. Because of inadequate public education and lack of law enforcement for child-proof containers in China, caustic substances are dispensed and sold in ordinary bottles that can be easily opened by children, resulting in accidental caustic substance ingestion. The rate of esophageal stricture formation after caustic ingestion is reported to be between 2% and 63% [3–5].

Current management for CES includes esophageal dilatation, retrievable stent placement, surgical resection of short segment stricture, and esophageal replacement. The foremost goal of treatment for CES is to preserve the esophagus and restore its function. Dilatation has been considered as the treatment of choice for CES and can be performed endoscopically or fluoroscopically, using a balloon dilator or rigid dilator [6]. The major disadvantage of fluoroscopically guided dilatation is repeat exposures to radiation because of the requirement for multiple dilatation treatment sessions. Several case series reports have shown that endoscopic balloon dilatation (EBD) is a safe and effective treatment for children with CES [7, 8]. However, there is still a lack of a well-established consensus on when and how to optimize dilatation in children with CES. Esophageal stent placement or surgery is indicated when dilatation is not successful. In our center, the prerequisite for esophageal stent placement is that the diameter of a stricture can be dilated at least to 1 cm, which is the smallest stent diameter available commercially. Surgery is usually performed when dilatation fails, esophageal perforation occurs with dilatation, or stent placement is contraindicated. Gastric tube esophagoplasty (GTE) has become the most commonly used operation for esophageal replacement in pediatric surgery [9, 10]. The aim of this study was to evaluate the long-term outcomes of endoscopic balloon dilatation (EBD) in treating CES in children and the influencing factors associated with EBD treatment outcome.

## 2. Patients and Methods

A retrospective case-series analysis was carried out in a tertiary children's hospital in Guangzhou, China. We included all patients who had a diagnosis of CES and underwent EBD from August 1, 2005, to December 31, 2014. Patients were followed up until December 31, 2015. We excluded patients who had previous dilatation or surgery for stricture in other hospitals or who were lost for follow-up. The medical records were reviewed to obtain each patient's demographic and clinical data including age, gender, grade of dysphagia, stricture site and length, stricture diameter, type of caustic substance, type and number of treatments, the date and duration of treatments, and adverse events.

Dysphagia was graded according to the patient's ability to swallow at initial presentation: 0, no dysphagia; 1, intermittent solid food dysphagia; 2, unable to swallow solids; 3, unable to swallow pureed food; and 4, unable to swallow liquids [6].

EBD is considered the treatment of choice for CES [6–8]. An esophageal stent placement is indicated for refractory esophageal stricture [11], which refers to those that do not respond to repeated esophageal dilatations at an up to 4-week interval and continues to anatomic stricture and persistent dysphagia [12]. Surgery is indicated if a severe esophageal stricture was not suitable for dilatation or refractory to repeated EBD and/or stent placement, or if esophageal perforation occurred during dilatation [13].

### 2.1. Endoscopic Balloon Dilatation

Initial dilatation was performed at ≥2 weeks after caustic ingestion. Dilatation was repeated every 2 to 4 weeks in the first few months and as needed depending upon the degree of dysphagia. Various types of gastroscopes (standard or high-definition, with 4.9–9.8 mm diameters; Olympus, Fujinon, or Pentax), balloon dilators (Boston Scientific, USA), and force pump (Alliance, Boston Scientific, USA) were used.

Under general anesthesia with endotracheal intubation, the gastroscope was inserted and advanced just above the stricture area. A balloon dilatation catheter (Boston Scientific, USA) of appropriate size (6–18 mm) was chosen according to the initial endoscopic estimation of stricture or previous dilatations and inserted and inflated for 2 minutes, followed by deflation for 2 minutes and repeated inflation for 2 times based on our own experience and others [14]. For strictures longer than 5 cm, dilatation begun from the upper section and then moved down to the lower section. For a stricture diameter less than 5 mm after dilatation, a nasogastric tube was inserted to prevent esophagus closure. Antibiotics were routinely administered for 48 h after the procedure.

### 2.2. Esophageal Stent Placement

Under general anesthesia with endotracheal intubation, the stricture was dilated to 1 cm and then a guide wire was inserted through the scope's biopsy channel and pushed out the scope. A stent conveyer was inserted through the guide wire and positioned using a standard protocol. With the supplied delivery system, a stainless steel stent (Sigma Jiangsu, China; type Z with diameter 1–1.4 cm) was placed. Endoscopy was performed after stent placement to make sure that the upper side of the stent was 2 cm above the stricture margin. Routine X-ray was used to check for stent migration every 2 weeks. Antibiotics were routinely administered for 48 h after the procedure. The stent was left in place for up to 3 months.

### 2.3. Surgery

Gastric tube esophagoplasty (GTE) was performed for long stricture whereas a narrow segment resection was performed for short strictures [13].

### 2.4. Outcomes

The primary outcome of this study was EBD success, which was defined as the maintenance of dysphagia-free status for at least 12 months after last EBD. EBD failure was defined as persistent or worsening dysphagia after EDB or requirement for further interventions including stent placement and/or surgery. The secondary outcomes included influencing factors associated with EBD success, serious adverse events such as perforation, bleeding, infection, mortality, and the percentage of patients requiring stent placement and/or surgery.

### 2.5. Statistical Analysis

The SPSS version 24.0 (IBM SPSS Statistics for Windows; IBM Corporation, Somers, NY) was used for data analysis. Categorical data were expressed as percentages and compared by using the Fisher exact test or the chi-square test. Continuous variables were expressed as mean, standard deviation, range, maximum, and minimum, based on the data characteristics. Quantitative data were assessed for normality by the Kolmogorov-Smirnov test and compared by using the Student *t*-test, and abnormality quantitative data was compared with the rank-sum test. Because treatment variable (EBD or surgery) is categorical data, and the same subject might had more than one treatment, a recently available generalized linear mixed model procedure was applied to include both fixed effects and a random effect to account for within-subject correlations due to repeated observations of the same patients. This analysis was used to fit the multilevel logistic regression model to our data with hierarchical structure.

## 3. Results

### 3.1. Patients

The institutional ethics committee of Guangzhou Women and Children's Medical Center approved this study protocol (approval number 2017102414).

A total of 46 patients met inclusion criteria, but 3 patients were excluded due to loss to follow-up. Thus, 43 patients (29 males) were included for analysis (Table 1). Mean age at first dilatation was 44 months (range 18–121 month). Only 3 patients were admitted to our hospital immediately after caustic substance ingestion, and all others were referred to our hospital for dysphagia after the acute phase. The mean time for referral was 49.7 days after caustic ingestion (range 1–140 days).

As shown in Table 1, dysphagia scores of patients were as follows: grade 1 (*n* = 1), grade 2 (*n* = 11), grade 3 (*n* = 30), and grade 4 (*n* = 1). The lengths of the strictures, as assessed by endoscopic and/or radiologic measurements, were as follows: 9 (20.9%) patients ≤2 cm and 34 (79.1%) patients >2 cm. Locations of esophageal strictures were in the upper esophagus in 4 (9.3%), in the middle esophagus in 31 (72.1%) patients, in the lower esophagus in 3 (7.0%) patients, in the middle-lower esophagus in 4 (9.3%) patients, and in the whole esophagus in one case (2.3%).

### 3.2. Endoscopic Balloon Dilatation (EBD)

**Figure 1:**
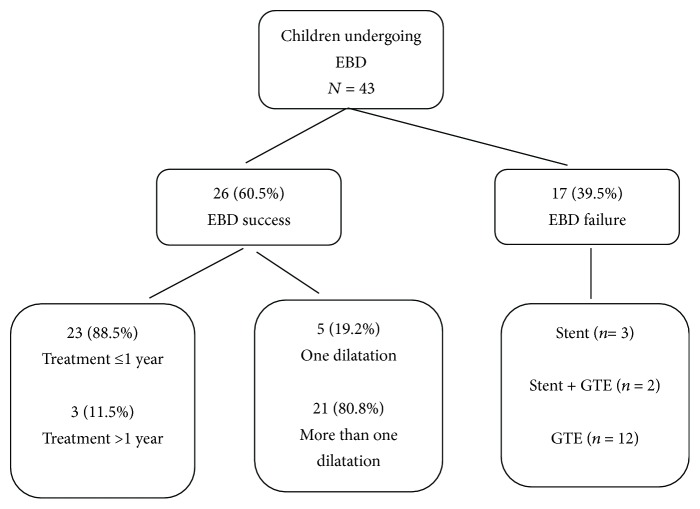
Flowchart of treatment outcomes of children with caustic esophageal strictures. EBD: esophageal balloon dilatation; GTE: gastric tube esophagoplasty.

A total of 168 EBD sessions were performed in 43 patients (Figure 1). Twenty-six (60.5%) patients were considered EBD success. 17 (39.5%) patients were considered EBD failures and underwent stent placement and/or surgery. In the EBD success group, 23 (88.5%) patients achieved dysphagia-free status within 12 months and another 3 (11.5%) patients achieved dysphagia-free status between 12 to 18 months; 21 (80.8%) patients required more than one dilatation, and 5 (19.2%) patients required only one dilatation.

As shown in Table 2, univariate analysis showed that patients in the EBD failure group had significantly longer stricture segments when compared to the EBD success group (*t* = 4.622, *P* < 0.001). Between the two groups, there were no statistical differences in stricture diameter, time of first EBD, number of EBD, and interval between EBD sessions. No differences were observed among different caustic substances (*χ*^2^ = 0.251, *P* = 0.999). In addition, effectiveness of EBD treatment did not differ among different dysphagia scores (*χ*^2^ = 2.125, *P* = 0.999). In multivariate analyses with generalized linear mixed models, the length of stricture in the EBD failure group was higher than that in the EBD success group (*t* = 2.398, *P* = 0.018, OR = 3.206, 95% OR: 1.228–8.371), but other variables were not associated with the different treatment outcomes.

Seven (4.4%) esophageal perforations occurred in 6 (13.6%) patients. No dilatation-related mortality, massive hemorrhage, or severe infections were observed. Four patients were managed initially with fasting, intravenous antibiotics, and parenteral nutrition, and two patients required gastrostomy placement. After 10–14 days of treatment, radiologic studies revealed perforation closure but esophageal strictures were severe and long (4–10 cm). All six patients had GTE later.

### 3.3. Esophageal Stent Placement

Five stents were placed in 4 patients because of persistent dysphagia after repeated EBD (8, 10, 12, and 15 times, resp.). All 4 patients had long segment strictures. Two patients ingested alkali and two ingested acids. Stents were removed after 2–3 months. Three patients had clinical resolution after stent placement 1-2 times. Two patients failed stenting and subsequently had surgery.

### 3.4. Gastric Tube Esophagoplasty (GTE)

GTE was performed in 14 patients. All of them had long strictures. Nine patients ingested alkali and 5 ingested acids. Two patients developed an anastomotic fistula which closed after 10–14 days of conservative management including intravenous antibiotics and nasogastric feeding. Two patients developed an anastomotic stricture which resolved after EBD 1-2 times.

## 4. Discussion

Esophageal balloon dilatation has been recommended as the choice of treatment for esophageal stricture in children. Patients with caustic substance-induced strictures have less satisfactory outcome and require a significantly higher number of dilatation sessions for improvement as compared with noncaustic strictures [3, 4, 6–8, 15]. However, there is still lack of a well-established consensus on when and how to optimize dilatation in children with CES, such as the best time to begin dilatation, the dilatation interval, the maximum number of dilatations to be considered failure, and the time to consider other treatment options. In our series, 60% of patients had clinical resolution with EBD and 40% failed EBD. Only length of stricture segment was statistically different between the EBD success group and the failure group. Initial dysphagia scores were not associated with the outcome of EBD. We did not observe any difference in time of the first EBD between EBD clinical resolution and failure groups.

The EBD success rate of 60% in our study is lower than that reported by others [8, 16] and may be due to several factors: (1) Most of our patients had long segment strictures. (2) Most of our patients were referred to our hospital more than 4 weeks after caustic substance ingestion. Uygun et al. [16] reported that earlier (7–25 days after ingestion of caustic substances) balloon dilatation was more effective with significantly shorter dilatation duration and less dilatation sessions when compared to late (26–60 days) dilatation. Alshammari et al. [8] waited at least 3 weeks between EBDs. A decision to consider balloon dilatation as failure is still debatable. Some studies suggest that esophageal strictures require 6 months to 3 years for stabilization [17–19]. In our study, the interval in EBD was between 3 and 40 weeks with an average of 8 weeks. In our EBD success group, the majority of patients (88.5%) were resolved within the first year.

With repeated EBD, 40% of our patients did not have clinical resolution and required stent placement and/or GTE. In our study, 7 (4.4%) esophageal perforations occurred in 6 (13.6%) patients, which is consistent with the observation reported by Contini et al. [18], who showed that delayed dilatation (>6 weeks after caustic ingestion) in children carried a higher risk of perforation and a higher recurrence rate in comparison to timely (<6 weeks) dilatation.

In conclusion, we report here our experience of 43 children with caustic esophageal stricture treated with EBD in the past ten years. Twenty-six (60.5%) patients considered EBD success, which correlated with stricture length. However, about 40% of our patients did not have clinical improvement after EBD and required stent placement and/or GTE. Optimization of EBD is needed for the treatment of caustic esophageal stricture in children.

## Figures and Tables

**Table 1:** Characteristics of patients with caustic esophageal stricture.

Variable	Statistics
Total number of patients	43
Boy	29 (67.4%)
Girl	14 (32.6%)
Mean age (range)	44 months (18–121)
Dysphagia score	
Grade 1	1 (2.3%)
Grade 2	11 (25.6%)
Grade 3	30 (69.8%)
Grade 4	1 (2.3%)
Length of stricture (cm)	
Short stricture (≤2 cm)	17 (39.5%)
Long stricture (>2 cm)	26 (60.5%)
Stricture location	
Upper esophagus	4 (9.3%)
Middle esophagus	31 (72.1%)
Lower esophagus	3 (7.0%)
Middle and low esophagus	4 (9.3%)
All esophagus	1 (2.3%)

**Table 2:** Comparison between esophageal balloon dilatation (EBD) clinical resolution and failure groups. Values are expressed as mean ± SD (range).

Variable	EBD success *N* = 26mean ± SD (range) or N	EBD failure *N* = 17mean ± SD (range) or *N*	Univariate analysis		Multivariate analyses (generalized linear mixed models)				95% OR LCI	95% OR UCI
			Statistics	*P*	Coefficient	t	*P*	OR		
Diameter of stricture (mm)	3.4 ± 1.5 (6)	2.7 ± 1.2 (5)	1.69^a^	0.099	−0.24	−0.539	0.59	0.787	0.326	1.896
Length of stricture (cm)	4.0 ± 1.9 (7)	7.6 ± 3.2 (13)	4.622^a^	**<0.001**	1.165	2.398	**0.018**	3.206	1.228	8.371
Time of first EBD (weeks)	7.6 ± 3.3 (16)	6.6 ± 2.9 (12)	0.952^a^	0.347	0.045	0.491	0.624	1.046	0.872	1.033
Number of EBD	3.0 ± 1.8 (7)	5.2 ± 4.6 (15)	1.443^b^	0.149	—	—	—	—	—	—
Interval of EBD (months)	2.3 ± 2.3 (9.7)	1.9 ± 1.5 (4.9)	0.623^a^	0.536	−0.003	−0.009	0.993	0.997	0.445	2.182
Median follow-up time (months)	77.0 ± 27.0 (97)	64.0 ± 13.0 (44)	1.628^b^	0.104	−0.054	−1.235	0.219	0.948	0.87	1.033
Age	46 ± 29 (121)	40 ± 23 (83)	0.698^a^	0.489	−0.044	−0.449	0.654	0.957	0.787	1.163
Gender (female/male (reference))	7/19	7/10	0.951^c^	0.329	1.908	0.878	0.381	6.74	0.092	493.12
Caustic substance										
Alkali	16	10			−2.091	−0.849	0.397	0.124	0.001	16.025
Acids	5	4			1.154	−0.404	0.687	3.171	0.011	896.717
Detergents and bleach (reference)	5	3	0.251^c^	0.999	—	—	—	—	—	—
Dysphagia score										
Grade 1	1	0			−21,567	−0.003	0.999	0	0	—
Grade 2	7	4			−8.608	−0.003	0.999	0	0	—
Grade 3	18	12	2.125^c^	0.811	−10.309	−0.003	0.999	0	0	—
Grade 4 (reference)	0	1			—	—	—	—	—	—
Stricture location										
Upper esophagus	2	2			−18.338	−0.014	0.993	0	0	—
Middle esophagus	19	12			−21.503	−0.017	0.991	0	0	—
Lower esophagus	3	0	3.664	0.552	−21.61	−0.017	0.991	0	0	—
Middle and low esophagus	2	2			−32.553	−0.013	0.993	0	0	—
All esophagus (reference)	0	1			—	—	—	—	—	

^a^ *t*-test; ^b^rank-sum test; ^c^chi-square test or Fisher exact method. Values are expressed as mean ± SD (range) in EBD clinical resolution and EBD failure.

## Data Availability

The data supporting the current findings are not publicly available since the database is currently not anonymous and contains all the patients' names. However, it will be available upon request.
